# Fixation of platelet-rich plasma and fibrin gels on knee cartilage defects after microfracture with arthroscopy

**DOI:** 10.1007/s00264-022-05377-2

**Published:** 2022-04-08

**Authors:** Mingjun Wang, Wenxiang Gao

**Affiliations:** Luoyang Orthopedic Hospital of Henan Province & Orthopedic Hospital of Henan Province, No.100 Yongping Road, Zhengzhou, 450046 Henan Province China

**Keywords:** Arthroscopy, Knee joint, Cartilage diseases, Platelet-rich plasma, Osteoarthritis, Visual analogue scale

## Abstract

**Purpose:**

An investigation of arthroscopic surgery combined with coverage of the microfractured wound surface with platelet-rich plasma (PRP) and fibrin gels (FG) to treat knee cartilage defects.

**Methods:**

Between February 2017 and February 2020, 145 patients with knee cartilage defects were treated. Only isolated full-thickness cartilage defects were included, and 28 patients (12 men and 16 women) were included in this study. They were all treated with arthroscopic surgery on subchondral bones, filled with PRP and thrombin, and sealed with FG. The knee pain visual analogue scale (VAS) scores were measured after the patients climbed ten stairs up and down, and the Western Ontario and McMaster Universities osteoarthritis index and the area of cartilage defects were measured through the pre-operative and post-operative follow-up. The complication incidences were also observed.

**Results:**

All patients were followed up for ten to 15 months (median 12 months). The knee pain VAS scores decreased from 6.57 ± 1.07 pre-operatively to 2.09 ± 1.35 at the last follow-up. The WOMAC osteoarthritis index decreased from 44.32 ± 3.95 (mean ± sd) pre-operatively to 16.57 ± 2.20 by the last follow-up. The cartilage defect decreased from 2.93 ± 0.65 cm^2^ pre-operatively to 1.09 ± 0.69 cm^2^ at the last follow-up. All scores showed statistically significant improvements after surgery (*p* < 0.05). No complications were observed.

**Conclusion:**

The combination therapy of arthroscopic surgery and covering the microfractured wound surface with PRP and FG can repair knee cartilage defects, relieve pain, and improve function, and is a safe and effective treatment.

## Introduction

Platelet-rich plasma (PRP), as a carrier of a variety of natural bioactive factors, has received increasing attention in the treatment of knee cartilage defects in recent years [1–3]. The standard method of application is by injection into the knee joint cavity, but PRP spreads to the entire joint cavity, and the therapeutic effect is limited [4]. Microfractures can also be used to treat cartilage defects by stimulating bone marrow to promote cartilage production [5, 6]. Studies have shown that PRP injection combined with microfracture can enhance the repair response of cartilage defects [7]. However, there is no relevant report on the curative effect of fixing PRP on the wound surface after microfracture. Therefore, we conducted relevant clinical studies to investigate the outcomes of arthroscopic surgery combined with covering the microfractured wound surface with platelet-rich plasma (PRP) and fibrin gels (FG) for the treatment of knee cartilage defects.

## Materials and methods

Between February 2017 and February 2020, 145 patients with knee cartilage defects were treated in our hospital. This study included only patients with isolated full-thickness cartilage defects. The exclusion criteria were as follows: body mass index (BMI) > 35 kg/m^2^, kissing lesions, cartilage defect area > 4 cm^2^ or diameter > 2 cm, inflammatory or rheumatoid arthritis, ligamentous instability, meniscus injury, and coronal malalignment > 7°. Based on these criteria, 28 patients (12 men and 16 women) with knee cartilage defects, treated with limited fixation of PRP after microfracture, were included in this study. The mean age was 31.09 years (range 16 to 42 years), the mean time from injury or from feeling uncomfortable until the operation was five weeks (range 1 to 8 weeks), and the mean BMI was 22 (range 18 to 27). Twelve patients had cartilage defects in the medial femur condyle, six patients in the lateral femur condyle, seven patients in the patella, and three patients in the femoral trochlear. Among them, there were 19 cases with a clear history of trauma and nine cases without a clear history of trauma.

This study was approved by the hospital ethics committee. Informed consent was obtained from all patients involved in the study.

The curative effects were evaluated according to the pain visual analogue scale (VAS), after climbing up and down ten steps, and the Western Ontario and McMaster Universities (WOMAC) osteoarthritis index. The size of the cartilage defect was measured using magnetic resonance imaging (MRI).

### Surgical methods and post-operative management

All patients underwent arthroscopy and all of the operations were performed by the same surgeon. Patients with a local full-thickness defect of the knee articular cartilage ≤ 4 cm^2^ or a defect diameter < 2 cm were included in the study. The same experienced surgeon prepared the PRP according to the method described by Landesberg et al. [8]. Autologous venous blood (50 ml) was centrifuged at 200 g for ten minutes. The plasma was then transferred to another tube and spun again at 200 g for ten minutes. The upper half of the preparation was designated platelet-poor plasma and the lower half was the PRP. Approximately 4 ml of PRP was obtained, mixed well, and set aside.

After arthroscopic exploration and clean-up, the patients underwent microfracture of the subchondral bone under arthroscopy. A microfracture cone (Wuyang Medical Device Co., Ltd.) was used at the cartilage defect to produce lesions at a spacing of 3 mm, diameter of 2 mm, and depth of 2 to 5 mm, until exudation of bone marrow in the cancellous bone occurred. Negative pressure suction drained the water in the joint cavity, and the tourniquet was then loosened. Bone marrow and blood would exude from the microfracture and partially adhere to the cartilage defect. The tourniquet was then inflated again, and air was injected to expand the joint cavity to allow even distribution of the PRP and thrombin (Hunan Yige Pharmaceutical Co., Ltd.). The injection was applied to the surface defect and then fibrin gel (Guangzhou Beixiu Biotechnology Co., Ltd.) was used for sealing. The tourniquet was then loosened to observe the condition of the seal and ensure that the cartilage defect was firmly sealed by the fibrin gel.

All knees were fixed in the joint extension position for one week and the patients remained non-weight-bearing on crutches for six weeks. The weight-bearing was gradually increased from six to eight weeks. Eight weeks later, the patients started to walk with full weight and returned to complete unrestricted activities.

### Observation indicators

The VAS score of knee joint pain after the patient climbed ten stairs up and down was obtained before surgery and during the post-operative follow-up: the score ranged between “no pain” (score of 0) and “worst pain imaginable” (score of 10). Each patient’s WOMAC osteoarthritis index [9] was assessed before surgery and during the post-operative follow-up: pain, stiffness, and physical function were quantified using a total of 24 questions, with a Likert scale of 0 to 4; the highest (worst) scores give a total of 96 points. The area of cartilage defect at the same position was measured. All the images obtained from the MRI inspection were imported into the software of the Materialise interactive medical image control system (MIMICS) (S10.1, Materialise Co., Ltd. Belgium) in Digital Imaging and Communications in Medicine (DICM) format, read and segmented, and the area of cartilage defect was calculated. Complications were checked during the treatment and at each follow-up.

### Statistical analysis

The statistical software SPSS (version 20.0) was used to compare pre- and post-operative results with paired *t*-tests. Differences were considered statistically significant at *p* < 0.05.

## Results

All patients were followed for ten to 15 months, with a median of 12 months. No complications were reported during the treatment or follow-up.

At the last follow-up, the knee joint pain after the patient climbed up and down ten stairs was significantly relieved, and the VAS scores were lower than those before treatment. The knee pain VAS scores decreased from 6.57 ± 1.07 pre-operatively to 2.09 ± 1.35 at the last follow-up. The differences in pre- and post-operative VAS scores were statistically significant (*p* < 0.05).

At the last follow-up, the WOMAC osteoarthritis index was lower than before treatment. The average pre-operative index was 44.32 ± 3.95, and the average post-operative index was 16.57 ± 2.20. The differences between the pre- and post-operative index were statistically significant (*p* < 0.05).

**Fig. 1:**
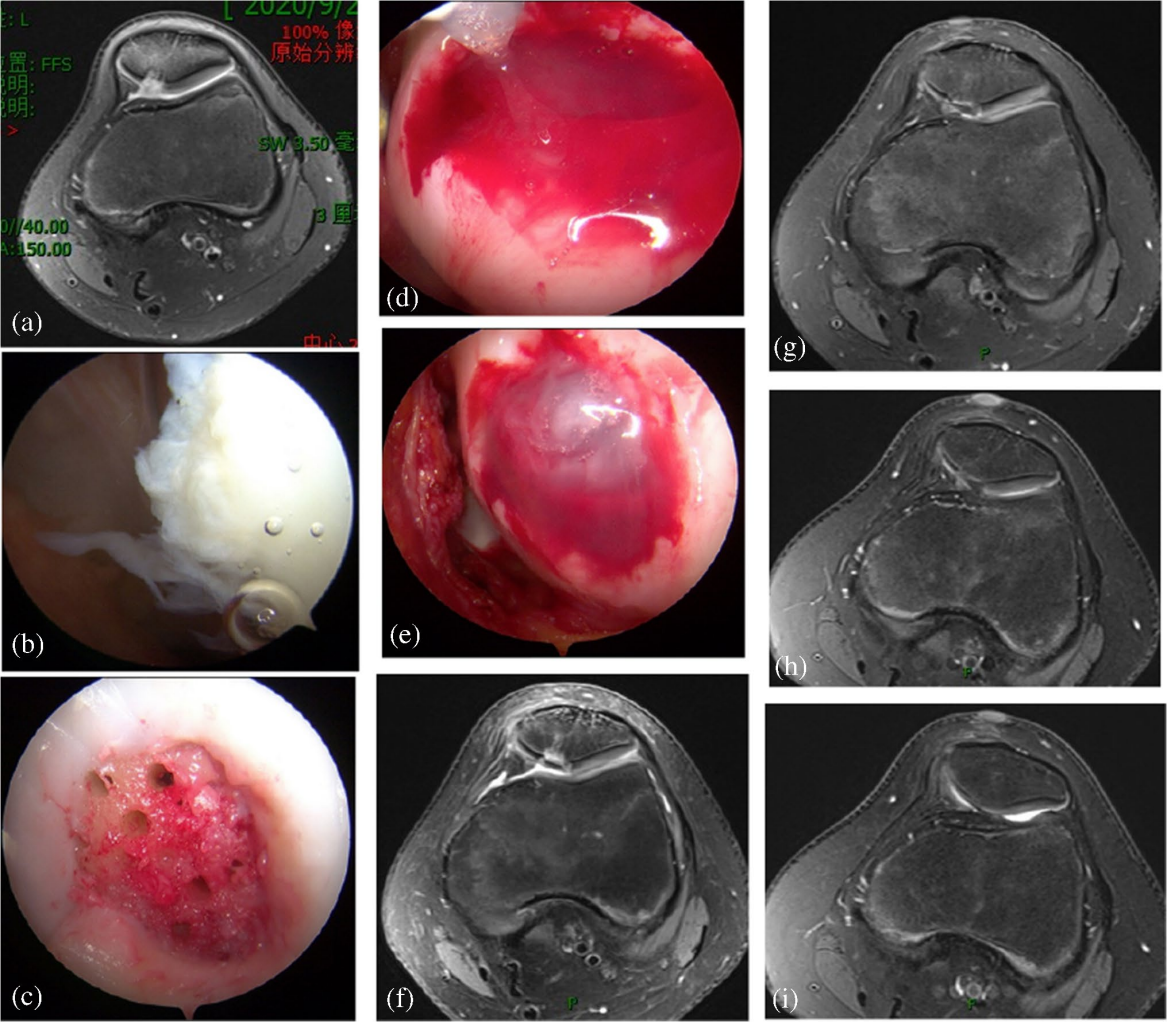
Images from typical cases. **a** The pre-operative MRI of the knee joint showed a full-thickness cartilage defect of the patella of approximately 2 cm^2^. **b** The full-thickness cartilage defect of the patella under arthroscopy. **c** A microfracture of the subchondral bone was created in the cartilage defect by using a microfracture device under arthroscopy. **d** Platelet-rich plasma and thrombin cover the microfracture wound. **e** The platelet-rich plasma and thrombin were sealed with fibrin gels. **f** Two months after surgery, the defects of the patella cartilage showed on the MRI. **g** Five months after surgery, the defects of the patella cartilage showed on the MRI. **h** Eight months after surgery, the defects of the patella cartilage showed on the MRI. **i** One year after surgery, the magnetic resonance imaging showed that most of the defects of the patella cartilage were repaired, and the area reduced to about 0.4 cm^2^

At the last follow-up, the cartilage defect area had decreased. The average pre-operative cartilage defect area was 2.93 ± 0.65 cm^2^, and the average post-operative cartilage defect area was 1.09 ± 0.69 cm^2^; these results were statistically significant (*p* < 0.05). Representative images of the procedure and outcomes are shown in Fig. 1.

## Discussion

Microfracture is a method of drilling the subchondral bone evenly and vertically to form a rough surface, which allows a haematoma to adhere and fill the defect. Potential stem cells infiltrating the bone marrow can differentiate into fibrochondrocytes and repair cartilage defects [5, 10]. Namdari et al. [11] treated 24 American Basketball Association athletes for knee cartilage injuries using microfractures and conducted follow-up observations; 14 athletes could return to the game. For patients with femoral condyle cartilage defects of < 4 cm^2^, the microfracture technique usually has a good clinical effect; for larger defects, the effect is often poor [12, 13]. Therefore, we selected patients with knee articular cartilage defects with an area ≤ 4 cm^2^ or diameter ≤ 2 cm in this study, and the defect area decreased to < 2 cm^2^ after treatment.

After PRP is activated, α particles can release a large number of cell growth factors, such as vascular endothelial growth factor, epidermal growth factor, platelet-derived factor, basic fibroblast growth factor, and transforming growth factor-1 [14]. PRP not only stimulates chondrocyte proliferation and matrix secretion but also inhibits the expression of inflammatory factors and chondrocyte apoptosis [15]. The high concentration of PRP can repair the degenerated articular cartilage of rats with knee osteoarthritis (KOA) to a certain extent and improve exercise ability [16]. At present, the clinical application of PRP to treat KOA is mainly by intra-articular injection [17], but PRP spreads to the entire joint cavity and affects the treatment [18]. Many technologies have been developed to improve the quality of cartilage repairs, such as microfracture combined with I/III collagen matrix-mediated cartilage regeneration technology [19], microfracture combined with absorbable PGA-hyaluronic acid implant, or platelet-rich fibrous gel covering [20]. Based on the above research, we used PRP, which can release a variety of growth factors, to cover the cartilage defect. These factors stimulate the potential stem cells that infiltrate from the bone marrow after microfracture to differentiate into fibrochondrocytes, thereby promoting cartilage repair.

Fixing PRP on cartilage defects with FG is also a key factor for better treatment effects. FG has been used in joint surgery, such as the repair of osteochondral damage and autogenous cartilage transplantation [21, 22]. Fibrinogen and thrombin can be mixed to immediately form a jelly, which can adhere to the cartilage defect and adopt a shape consistent with the defect. The fibrous gel can be used to seal cartilage or osteochondral defects and can be firmly attached to the defect [21, 23]. However, experiments on cadaver specimens have shown that knee movement causes allogeneic bone particles filled in the cartilage defect to be displaced immediately after being sealed with fibrous gel. Therefore, it is recommended to limit the knee exercise after the graft is used to fill the cartilage defect [24]. Therefore, we restricted the movement of the knee for one week after surgery.

Brennan et al. [25] conducted a follow-up assessment of the MRI of KOA patients. The results showed that the area of cartilage defects in the natural state will gradually progress over time, with an annual progression rate of 2.5%. In this study, the cartilage defect did not progress over time, but decreased significantly. This suggests that the treatment scheme was effective. In this study, we used the conventional MRI standard sequence to detect the cartilage defect area to and evaluate the cartilage defect, which is the objective index of the clinical treatment effect of knee osteoarthritis. With the rapid development of MRI cartilage imaging technology, newer and more accurate technologies can provide quantitative information on cartilage biochemical status [26], which can improve the accuracy of assessment of non-full-thickness cartilage defects. However, the new technologies reported in the literature are not widely clinically applied, so the actual effect has yet to be tested by time. Therefore, conventional techniques and standard sequences are still the main detection methods in clinical practice. Studies have shown that the area of cartilage defects found during debridement of the degenerative arthritis joint is often significantly larger than the pre-operative MRI measurement [27]. However, these related studies mainly compared the results of the arthroscopic evaluation with pre-operative MRI test data [28]. At the same time, studies have shown that the integrity of the cartilage defect repair is consistent with the results of conventional MRI examination when the cartilage defect repair is inspected by arthroscopy [29]. Therefore, MRI is accurate for the detection of cartilage damage and bone marrow oedema in KOA [30]. In this study, we used MRI before and after surgery to evaluate the therapeutic effect of the surgery by comparing the changes in the area of cartilage defects in the same patient and at the same site, thus avoiding the error between arthroscopic and MRI measurements.

The results of this study suggest that when arthroscopic microfracture surgery is combined with PRP and FG to cover microfracture wounds cartilage defects can be repaired, thus reducing knee joint pain symptoms and improving knee joint function. This technique has high safety and excellent efficacy. Knee cartilage defects can be treated using simple microfracture surgery. However, due to the small sample size and short follow-up time, the results obtained need to be confirmed by further studies.

### Conclusion

Surgery using fixation of PRP with FG sealant on cartilage defects after microfracture can stop further aggravation of the knee defects, improve knee function, and decrease cartilage defects. This procedure is minimally invasive and an effective treatment for cartilage defects.

## Data Availability

All the data and materials are available.

## Code availability

The statistical software SPSS (version 20.0).
